# Evaluating the effect of chronic and continuous use of methadone on the glomerular filtration rate of patients receiving oral methadone (syrup)

**DOI:** 10.3389/fmed.2024.1496505

**Published:** 2024-12-19

**Authors:** Valiollah Goudarzvand Chegini, Amir Mohammad Kazemifar, Amir Ershad Tavakolian, Arian Ghannadi Karimi, Arsalan Jamali Pour Sofi, Darya Ipchian, Sahand Kamali, Mehran Ebrahimi Varkiani, Zohreh Yazdi

**Affiliations:** ^1^Department of Nephrology, Velayat Hospital, Qazvin University of Medical Sciences, Qazvin, Qazvin, Iran; ^2^Professor of Clinical Toxicology and Forensic Medicine, Qazvin University of Medical Sciences, Qazvin, Qazvin, Iran; ^3^Qazvin University of Medical Sciences, Qazvin, Qazvin, Iran; ^4^Preclinical Core, Cardiovascular Imaging Core Facility, Tehran University of Medical Sciences, Tehran, Tehran, Iran; ^5^Metabolic Disease Research Center, Qazvin University of Medical Sciences, Qazvin, Qazvin, Iran

**Keywords:** methadone, opioid dependence, glomerular filtration rate (GFR), nephrotoxicity, methadone maintenance therapy (MMT)

## Abstract

**Introduction:**

This study systematically examines the effects of chronic oral methadone use on the glomerular filtration rate (GFR) in patients participating in methadone maintenance therapy (MMT) in Qazvin City, Iran. Methadone, a synthetic *μ*-opioid receptor agonist, is predominantly utilized for the management of opioid dependence and pain relief; however, there is growing concern regarding its potential nephrotoxic effects.

**Methods:**

An observational cross-sectional study was executed involving 150 participants who had been on methadone syrup for a minimum duration of 2 years. Comprehensive data pertaining to demographic variables, methadone dosage, and serum creatinine levels were meticulously gathered at baseline, as well as at 3 and 6 months. GFR was calculated utilizing the Cockcroft-Gault formula.

**Results:**

The results demonstrate that, while the mean GFR values remained within the normal range, a significant correlation was observed between methadone dosage and a reduction in GFR; specifically, higher dosages were associated with lower GFR values. In contrast, the duration of methadone use did not significantly affect GFR.

**Conclusion:**

These findings indicate that, although methadone dosage may influence renal function, long-term methadone therapy does not inherently exacerbate the risk of chronic kidney disease (CKD) in this population. This underscores the critical need for diligent monitoring of methadone dosages to mitigate the risk of potential renal impairment and highlights the importance of further research into the long-term implications of methadone on renal health.

## Introduction

Methadone, a synthetic *μ*-opioid receptor agonist, is extensively employed in the management of opioid dependence (1). Methadone, derived from diphenyl heptanes (heptamines), exhibits significant efficacy as a clinical pain reliever. Its mechanism of action involves inhibiting pain impulse transmission by binding to opioid receptors in the spinal cord. Oral, intravenous, and subcutaneous routes can be utilized for the administration of methadone, which exhibits efficient absorption through the digestive system. As a result, its blood concentration exceeds that of oral morphine (2). Methadone is distributed extensively throughout the body, reaching various tissues, and it can cross the placenta. The onset of its effects is observed 30 to 60 min after oral ingestion and 10 to 20 min after intramuscular or subcutaneous injection. Metabolism of this drug occurs in the liver and is excreted from the body via urine (2, 3). Methadone proves beneficial in managing resistant cancer pain and neuropathic pain, particularly in cases where previous treatment with morphine has been ineffective. Consequently, when patients experience intolerable side effects from escalating doses of morphine or hydromorphone, transitioning to methadone treatment offers sufficient pain relief with only 10 to 20 % of the daily morphine dosage (1, 4). Methadone can lead to various side effects such as heat intolerance, dizziness, fainting, weakness, chronic fatigue, sleep disturbances, digestive issues, low blood pressure, constricted pupils, dry mouth, headaches, urinary problems, itching, decreased libido, euphoria, and respiratory complications (2, 5). The use of opioids can adversely affect kidney function by reducing kidney plasma flow. Additionally, opioids have been associated with the occurrence of acute kidney failure, nephrotic syndrome, and focal segmental glomerulosclerosis. These conditions can have long-lasting consequences for patients, manifesting as irreversible side effects (3, 6). In Iran, the management of opioid dependence has transformed significantly over the past few decades, evolving from punitive measures to comprehensive medical treatment strategies. Following the 1979 Islamic Revolution, aggressive criminal justice approaches were initially employed, which led to negative outcomes for those struggling with addiction. By the mid-1990s, the government recognized addiction as a disease, prompting the establishment of voluntary treatment centers that provided psychosocial support and non-agonist medications. The introduction of Methadone Maintenance Therapy (MMT) in the early 2000s was a critical development in Iran’s harm reduction efforts, with methadone being widely dispensed across numerous clinics to combat rising HIV rates among people who inject drugs. Today, Iran operates one of the largest MMT programs globally, serving over 300,000 patients while integrating harm reduction initiatives such as needle exchange programs. Despite these advancements, challenges like social stigma and access issues continue to hinder effective treatment for opioid-dependent individuals in the country (7, 8). The authors focus on the relationship between chronic methadone use and glomerular filtration rate (GFR) due to increasing concerns about nephrotoxicity in patients on methadone maintenance therapy. Long-term opioid use, including methadone, has been associated with renal complications such as acute kidney injury and chronic kidney disease, often linked to factors like rhabdomyolysis and altered renal blood flow. Understanding how prolonged methadone use affects GFR is essential for assessing the risk of renal impairment and guiding appropriate monitoring and management. As methadone is widely used for treating opioid dependence, clarifying its impact on kidney function is crucial for optimizing treatment outcomes and ensuring patient safety. This study aims to provide valuable insights into the long-term effects of methadone therapy on kidney health. Due to the significance of this issue, we decided to investigate the effects of chronic and continuous methadone use on the glomerular filtration rate (GFR) in patients who are administered oral methadone.

## Methods

**Figure 1:**
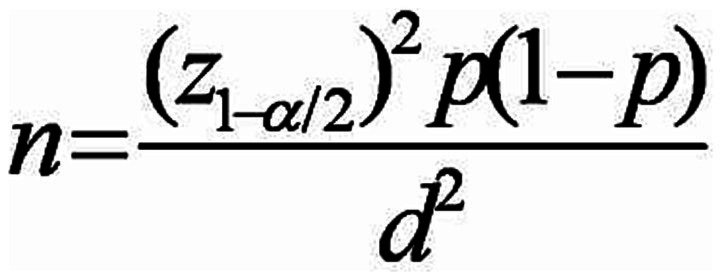
Statistical population calculation equation.

This observational study, conducted as a cross-sectional study, aimed to investigate 150 chronic and continuous users of methadone in addiction treatment centers in Qazvin City. The study was conducted for 3 and 6 months, during which demographic information was collected using a data collection form. The research was presented to the Qazvin University of Medical Sciences Medical Ethics Committee with ethics code number IR.QUMS.REC.1400.136. After receiving the introduction letter from the research vice-chancellor of Qazvin University of Medical Sciences, it was referred to the research unit, and then participants were given written questionnaires. Their consent was obtained by returning the completed consent forms with signatures and fingerprints. The participants in this research were selected based on meeting the inclusion criteria and not meeting the exclusion criteria. Data on methadone use duration, dosage, age, and creatinine levels were collected at entry as well as at 3 months and 6 months post-entry. The collected data was analyzed using SPSS 23 software, and the results were presented in a statistical table. The sample population for the study was determined using the sampling method. With an alpha value of 0.05, an accuracy of 0.05, and a prevalence rate of 0.1 for GFR disorder from the pilot study, 150 patients were included in the analysis (9, 10) (Figure 1).

To ensure the accuracy and reliability of the study, it is crucial to rule out certain chronic and systemic diseases, such as diabetes and high blood pressure, as well as the effects of nephrotoxic drugs like NSAIDs. Therefore, specific tests were conducted before enrolling patients in the study. These tests include serum FBS, U/A, CPK, and Cr tests. By doing so, we minimized errors and obtained more accurate results. Additionally, several measures were taken to ensure the validity of the study:

1. All experiments were meticulously recorded in a laboratory setting from the beginning to the end.
2. A comprehensive questionnaire was administered to gather information about the participant’s medical history, drug usage, and any significant events that may occur during the study. This includes contracting new or pandemic diseases like Coronavirus and using new medications, dosage, duration, and other relevant factors.
3. Methadone consumed by the participants was in the form of fixed-brand syrup (Darou Pakhsh) in three dosages 5 cc, 10 cc, 15 cc each contains 25 mg, 50 mg, 75 mg methadone, respectively.
4. Regular monitoring of serum creatinine level was conducted before starting methadone, at 3 months and finally at 6 months.
5. All patients that were included in our study started using methadone 2 years prior to the study.

### Inclusion and exclusion criteria for the study

Inclusion Criteria: The study included patients who have been using methadone syrup on a chronic and continuous basis. Additionally, participants were required to have the capability to accurately calculate their Glomerular Filtration Rate (GFR).

Exclusion Criteria: Several exclusion criteria were established to ensure the integrity of the study’s findings. These criteria included:

1. Patients undergoing dialysis, specifically those diagnosed with End-Stage Renal Disease (ESRD).
2. Individuals currently using nephrotoxic medications, with a particular emphasis on Non-Steroidal Anti-Inflammatory Drugs (NSAIDs).
3. Patients presenting with hypertension, defined as having a blood pressure reading exceeding 140/90 mmHg.
4. Individuals with a GFR below 80, indicating compromised renal function.
5. Patients who developed systemic diseases during the course of the study, including but not limited to COVID-19 or other conditions that could adversely affect renal health.
6. Any systematic disease that could adversely affect renal health such as diabetes, hypertension, COPD.

**Figure 2:**
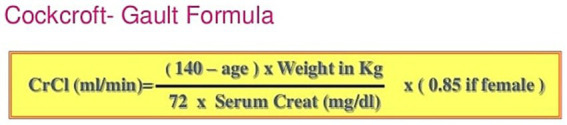
Cockcro8-Gault formula.

**Figure 3:**
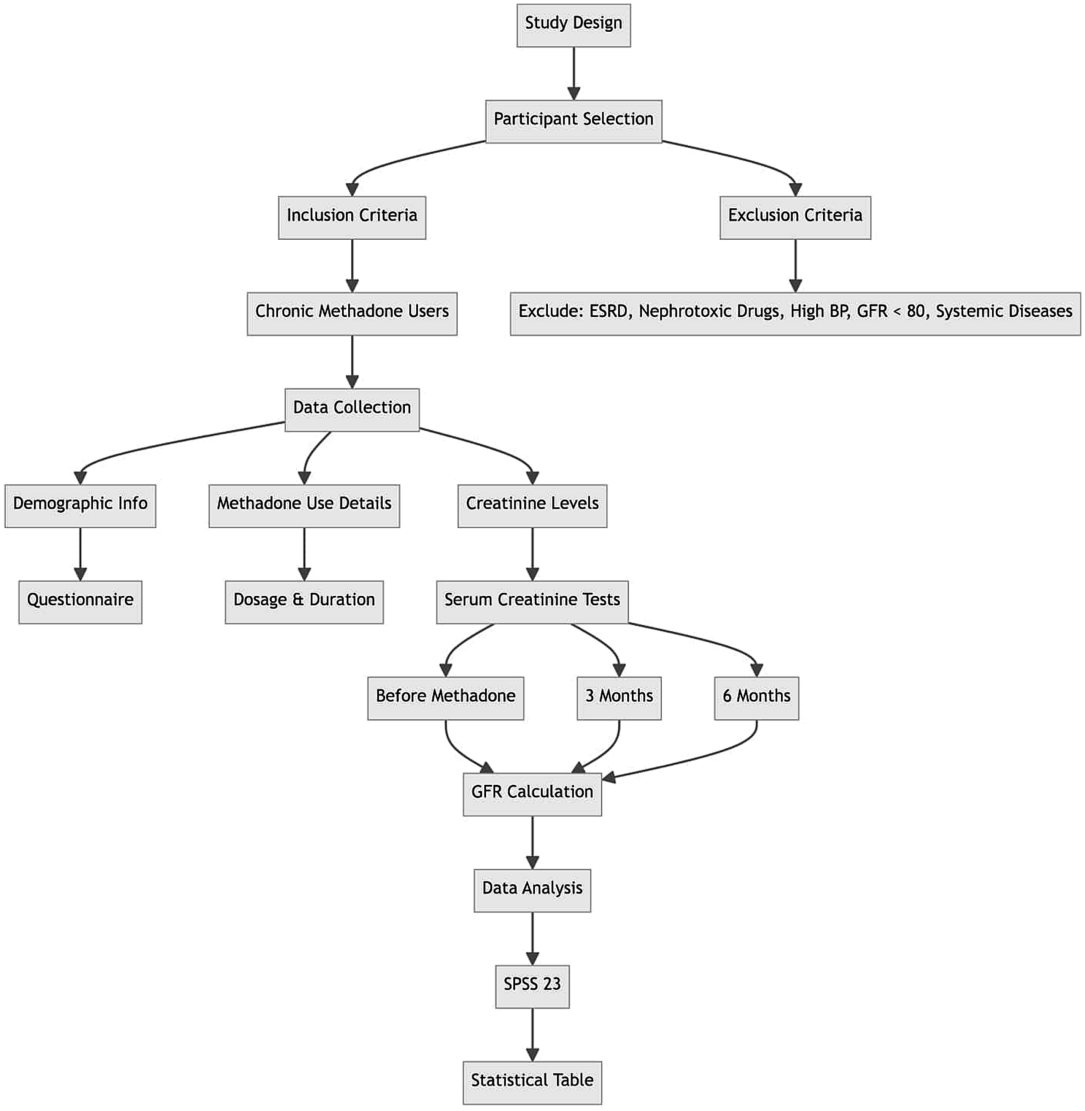
Demographic abstract of the study design.

The GFR in patients was determined using the Cockcroft-Gault formula based on laboratory results, particularly serum creatinine. This determination was made at the beginning, before starting methadone use, and then again at 3 months and 6 months into the use. The recorded data was collected in a form, and the frequency of GFR reduction was analyzed and compared based on variables such as age and gender, in the study participants (Figures 2, 3).

## Results

The results presented below are a result of the analysis of the collected data. The tables illustrate the age distribution, duration of consumption, and glomerular filtration rate at the time of referral, along with the measurements taken 3 and 6 months after referral. These findings are based on the mentioned statistical population and the collected information (Table 1).

**Table 1:** Glomerular filtration rate analysis at the time of referral and measurements taken 3 and 6 months after referral.

Glomerular filtration rate		Mean value	Standard deviation	Count
Referral time	5 (mg/day)	83/112	63/19	86
	10 (mg/day)	33/102	93/15	55
	15 (mg/day)	81/92	41/14	9
	Total	78/107	02/19	150
Three months after the referral	5 (mg/day)	46/112	7/19	86
	10 (mg/day)	56/104	06/16	55
	15 (mg/day)	51/91	45/15	9
	Total	31/108	18.97	150
Six months after the referral	5 (mg/day)	65/115	58/18	86
	10 (mg/day)	49/101	92/14	55
	15 (mg/day)	53/91	72/17	9
	Total	72/106	18.34	150

Additionally, the tables display the distribution of participants by gender and dosage frequency as presented in Tables 2, 3.

**Table 2:** The distribution of participants based on gender.

Variable	Number (Percentage)	
Gender	Male	132 (88)
	Female	18 (12)

**Table 3:** Participants are categorized according to their methadone dosage.

Variable		Number (Percentage)
Dosage (mg/day)	5	86 (57.3)
	10	55 (36.7)
	15	9 (6)

The statistical population’s mean and standard deviation at the initial testing and 3 and 6 months later (duration) subsequent tests are provided in Table 4.

**Table 4:** Statistical population’s mean and standard deviation at the initial testing and 3 and 6 subsequent tests.

Variable	Mean value ± standard deviation	Count
GFR at initial testing	02/19 ± 78/107	150
GFR 3 months after initial testing	97/18 ± 31/108	150
GFR 6 months after initial testing	34/18 ± 72/106	150

**Figure 4:**
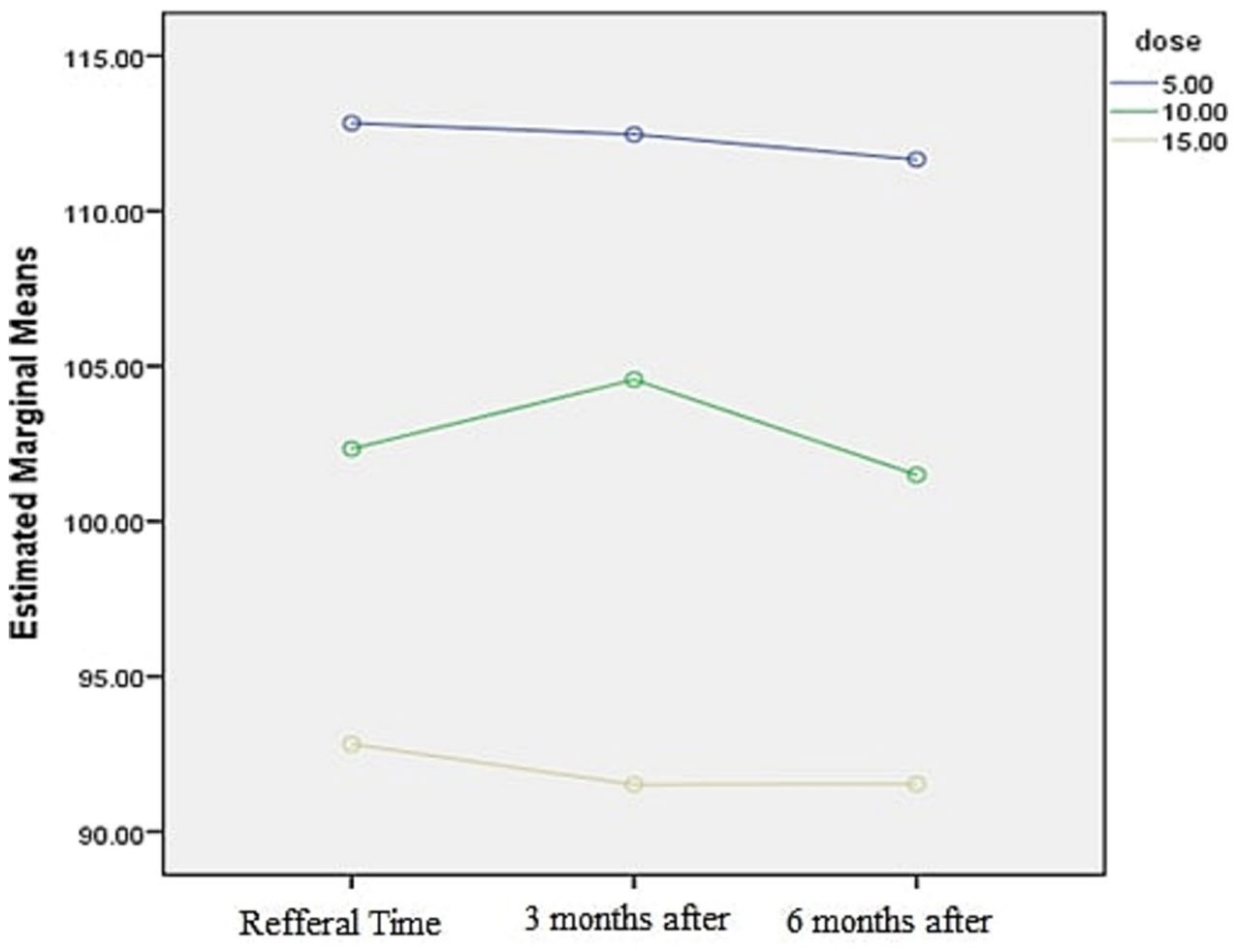
Demographic diagram of glomerular filtration rate.

The graph shows that the patient’s glomerular filtration rate (GFR) decreases as the dosage increases (Figure 4).

The glomerular filtration rate distribution was calculated using the Kolmogorov–Smirnov formula at the initial visit and at 3-and 6-months post-visit. The analysis revealed no significant relationship between duration of methadone use and glomerular filtration rate reduction (*p* value>0.05). Additionally, a substantial decrease in glomerular filtration rate was observed with dosage of consumption (*p* value>0.05)—the Sig. Mauchley’s test indicated a significant implementation method with a value of 0.0001 (Table 5).

**Table 5:** The average ± standard deviation glomerular filtration rate distribution across the recorded time intervals.

Duration dosage (mg/day)	Initial testing	Three months after the initial testing	Six months after the initial testing
5	112.83 ± 19.61	112.46 ± 19.70	111.65 ± 18.587
10	102.30 ± 15.93	104.56 ± 16.06	101.49 ± 14.92
15	92.817 ± 14.41	91.51 ± 15.45	91.53 ± 17.72
The repeated measures analysis of variance assessment	Duration effect	F: 0.883 Sig. 0.349	
	Dosage effect	F: 9.126 Sig. 0.0001	
	Duration-Dosage effect	Sig. 0.976	

## Discussion

This research aimed to assess the influence of long-term methadone usage on the GFR of patients who were administered oral methadone. A large proportion of the subjects were male (88%), and most of the participants’ methadone dose administration was 5 cc (25 mg; 57.3%). The mean GFR values for the patients at the time of examination, after 3 months, and after 6 months were documented as 107.78, 108.31, and 106.72, respectively.

The results of these studies demonstrated that the average glomerular filtration rate of the subjects was within the normal range at the time of the first visit, as well as at the 3-month and 6-month follow-ups. A significant relationship was observed between the duration of methadone use and the level of glomerular filtration. However, there was no significant correlation between the dose of methadone and the level of glomerular filtration. Extensive research on the long-term and continuous use of methadone and its effect on the glomerular filtration rate (GFR) of patients is currently lacking. The existing studies primarily consist of case reports, case series, or systematic review articles that mainly explore the renal side effects of methadone when combined with other drugs.

In a study by Ghasemi et al., a case of methadone poisoning was described following a suicide attempt with a 40 mg tablet of methadone. The patient developed rhabdomyolysis, acute renal failure, and bilateral sensorineural hearing loss. Interestingly, the hearing loss became a chronic effect of methadone, persisting despite kidney function and metabolic abnormalities normalization (11).

Metabolic acidosis and rhabdomyolysis induced by methadone can lead to acute kidney failure, necessitating hemodialysis. There have been limited reported cases of rhabdomyolysis following opioid poisoning. Various hypotheses have been suggested to explain the occurrence of rhabdomyolysis in opioid users, including dehydration, vascular insufficiency, muscle spasm, vasospasm, shock, trauma, convulsions, acidosis, respiratory failure, and direct effects of opioids. The occurrence of simultaneous rhabdomyolysis and hearing loss after opioid poisoning is exceedingly rare (12, 13).

In a comprehensive review conducted by Roy et al., various painkillers in renal failure patients were examined. The findings revealed that methadone and its metabolites do not accumulate in patients with CKD, eliminating the necessity for dose adjustment or dialysis. Interestingly, the study also observed that the GFR of CKD patients taking methadone did not decline over time. However, it is essential to note that methadone has the potential to prolong the QTc interval and, when combined with electrolyte imbalances commonly seen in kidney disease such as hypo/hyperkalemia or hypo/hypermagnesemia, may increase the risk of arrhythmias. As a result, the researchers recommended that methadone, as an analgesic, can be prescribed by any licensed healthcare provider and that patients are not required to attend a clinic with a licensed drug treatment program (14).

In the research conducted by Stalund and colleagues, it was reported that administering methadone oral syrup intravenously led to the accumulation of polyvinylpyrrolidone PVP (methadone metabolite) in patients with opioid addiction and intravenous drug use. Their results indicated a correlation between polyvinylpyrrolidone in kidney biopsies and renal tubule atrophy (15). Atherosclerosis was found to be a common occurrence in patients with a history of illicit drug use, as demonstrated in an autopsy investigation by Buettner and team, where methadone administration and metabolite deposition were linked to atherosclerosis. Additionally, Butner and colleagues documented interstitial fibrosis and tubular atrophy following methadone usage (16).

Two case reports from 1968 and 1972 have documented the presence of methadone metabolite deposition in the kidneys of patients with chronic kidney disease (CKD) (17, 18). In these cases, the methadone metabolite deposits were attributed to repeated injections of injectable drugs containing medium molecular weight methadone metabolites. Greenfield and colleagues conducted a study and observed that most glomerular methadone metabolite deposition occurred in podocytes. This deposition may be attributed to the lower molecular weight of the methadone metabolite previously used in injectable drugs. This could allow for partial glomerular filtration and potential reabsorption by the tubular epithelium (18).

Methadone-related syrup was available in six European countries from 2007 to 2014. Nevertheless, the intermediate molecular weight metabolite of methadone continues to be utilized as an excipient in various opioid substitution medications and other oral drugs with addictive properties in Europe and the United States. The repeated administration of such medicines could result in the accumulation of methadone metabolite to some extent (19).

In the Porubsky P et al. study, three patients exhibited proteinuria (20–2.5 grams per day) and kidney impairment. Kidney biopsies revealed evident extracellular and intracellular lipid substance depositions in glomerular, interstitial, and tubular regions. The researchers indicated that this renal lipidosis was associated with drug misuse, methadone usage, or intravenous misuse (20).

In the study conducted by Vallecillo G. et al., it was found that patients with heroin use disorder who were treated with methadone commonly exhibited overweight and metabolic syndrome. However, there was no mention of renal dysfunction or reduction in GFR, and no correlation was found between methadone dosage and GFR levels. The researchers highlighted a specific concern regarding the potential link between methadone exposure and metabolic syndrome (21).

## Conclusion

Methadone poisoning can lead to both acute and chronic kidney damage over time. While most cases of methadone toxicity result in temporary symptoms, there is a rare possibility of permanent kidney damage. Our research indicates that the dosage of methadone plays a role in reducing the glomerular filtration rate (GFR) and the development of chronic kidney disease (CKD). However, the duration of methadone use does not affect the GFR, suggesting that long-term methadone use does not pose a risk to kidney health. It is crucial to focus on fixing the methadone dosage rather than worrying about the duration of use.

### Limitation

1. Sample Size: The sample size may have been relatively small, which could affect the generalizability of the findings to a wider population of opioid users.
2. Confounding Variables: There may be confounding variables that were not adequately controlled for, such as co-occurring mental health disorders or concurrent substance use, which could influence the observed outcomes.
3. Follow-Up Duration: The follow-up duration may not have been long enough to capture long-term effects or adverse reactions associated with prolonged methadone therapy, potentially missing important aspects of patient health over time.

### Recommendation

We recommend conducting clinical studies to explore drug prevention strategies or ways to minimize the impact of methadone on kidney function in the long term. This will help prevent the occurrence of CKD and end-stage renal disease (ESRD).

## Data Availability

The original contributions presented in the study are included in the article/supplementary material, further inquiries can be directed to the corresponding author.
